# Supplementary material for: Extraintestinal Escherichia coli from camel carcasses: Phylogroups, serotypes, and markers of virulence
Source: PLoS One. 2025 Oct 9;20(10):e0334045. doi: 10.1371/journal.pone.0334045 (PMC12510483; doi:10.1371/journal.pone.0334045)
Supplement: Attachment — Submitted filename: Review.pdf [file pone.0334045.s006.pdf]

Reviewer's report

PONE-D-25-05895: Extraintestinal Escherichia coli from camel carcasses: Phylogroups, serotypes, and markers of virulence.

Escherichia coli has received increasing attention over the past decade as a cause of various intestinal and extraintestinal infections in humans and animals.
Infection with *E. coli* has serious consequences, such as growth retardation, weight loss and high mortality in farm animals, especially young animals. It is a major economic problem.
Extraintestinal pathogenic *E. coli* isolated from camel carcasses and fecal *E. coli* from healthy adult camels (as controls) were characterised by the authors of this manuscript for phylogenetic groups, O-serotypes and virulence-associated genes. Isolates from adult camels and young individuals from the same farm were compared. The researchers attempted to identify the source and transmission route of the bacteria by comparing fecal isolates from healthy camels as potential reservoirs of ExPEC infections.
The manuscript is written clearly and well organized but requires corrections.

Comments

In the **Abstract** and **Discussion,** there is a lack of opinion on the character of virulence factors. What is the significance of the virulence factors found? Not only the quantity but also the type of Vf is important, some belong to the so-called fitness factors, others indicate high virulence of the strains. The general role of Vf is tabulated, but this requires discussion.

The **Introduction** should describe the pathotypes of E. coli, particularly APEC, as the conclusion included information that APEC strains from wild birds may be responsible for infections.

Materials and methods

Details of the methodology sufficient to allow the experiments to be reproduced, but:

1. Only one isolate per sample was genetically tested, but I have doubts about whether this is a good approach. There may be several different *E. coli* genotypes with different genetic characteristics in one sample. At least 3-5 isolates with # phenotypes should be analysed. Clonal relationships can be excluded by genetic typing (e.g. PCR fingerprinting). Explanation is required.
2. In PCR, final concentrations in the reaction mixture should be reported, not volumes.

3. I understand that the same matrix was used in determining the phylogenetic group, serological group or Vf, there is no need to describe the method of DNA isolation for each PCR (L: 104 – 106; L: 121-123; L: 137-139)
4. L: 148 - pCVD432 is a large plasmid, according to me the information should be: pCVD432 gene/s

Results/ Discussion

1. L: 164-168 This information can be omitted, it is a repeat from Materials and Methods.
2. As a result of the research, it was found that the most common phylogenetic group was B1. This is contrary to the research results available in many scientific studies. The most pathogenic is group B2 and D. B1 belongs to the commensal group. It is worth discussing.
3. The significance of serotypes should be discussed. What do they indicate?
4. L: 199-200 I don't understand this information: "Desvaux et al., [23]) was assessed in **our** samples of camel pathogenic *E. coli*."
5. The discussion on virulence factors should be more specific. What are the consequences of the presence of these genes and what role could they play in pathogenesis? Only statistics are given without indicating which Vf are important.

I suggest a major revision.
